# Identification and Molecular Characterization of the Operon Required for L-Asparagine Utilization in *Corynebacterium glutamicum*

**DOI:** 10.3390/microorganisms10051002

**Published:** 2022-05-10

**Authors:** Koichi Toyoda, Riki Sugaya, Akihiro Domon, Masako Suda, Kazumi Hiraga, Masayuki Inui

**Affiliations:** 1 Research Institute of Innovative Technology for the Earth (RITE), 9-2 Kizugawadai, Kizugawa 619-0292, Japan; toyo51@rite.or.jp (K.T.); msuda@rite.or.jp (M.S.); khiraga@rite.or.jp (K.H.); 2 Division of Biological Science, Graduate School of Science and Technology, Nara Institute of Science and Technology, 8916-5 Takayama, Ikoma 630-0192, Japan; riki-sugaya.az@srigroup.co.jp (R.S.); chicharito14love@gmail.com (A.D.)

**Keywords:** asparaginase, aspartase, *Corynebacterium glutamicum*, L-asparagine, L-aspartate, transcriptional regulation

## Abstract

Understanding the metabolic pathways of amino acids and their regulation is important for the rational metabolic engineering of amino acid production. The catabolic pathways of L-asparagine and L-aspartate are composed of transporters for amino acid uptake and asparaginase and aspartase, which are involved in the sequential deamination to fumarate. However, knowledge of the catabolic genes for asparagine in bacteria of the Actinobacteria class has been limited. In this study, we identified and characterized the *ans* operon required for L-Asn catabolism in *Corynebacterium glutamicum* R. The operon consisted of genes encoding a transcriptional regulator (AnsR), asparaginase (AnsA2), aspartase (AspA2), and permease (AnsP). The enzymes and permease encoded in the operon were shown to be essential for L-Asn utilization, but another asparaginase, AnsA1, and aspartase, AspA1, were not essential. Expression analysis revealed that the operon was induced in response to extracellular L-Asn and was transcribed as a leaderless mRNA. The DNA-binding assay demonstrated that AnsR acted as a transcriptional repressor of the operon by binding to the inverted repeat at its 5′-end region. The AnsR binding was inhibited by L-Asn. This study provides insights into the functions and regulatory mechanisms of similar operon-like clusters in related bacteria.

## 1. Introduction

*Corynebacterium glutamicum* is a non-pathogenic, Gram-positive, and facultative anaerobic bacterium belonging to the class Actinobacteria. It has been used for the industrial production of amino acids, especially L-glutamate and L-lysine [1,2,3]. The annual global production of amino acids increased to 7.0 million tons in 2016 [4] and is expected to grow further. Strains for amino acid production have been routinely developed using mutagenesis and selection processes. Recent advances in systems and synthetic biology have enabled rational metabolic engineering of strains for the production of not only amino acids but also chemicals, alcohols, and proteins (reviewed in [4,5,6]). For a rational design, information pertaining to metabolic pathways, their regulation, and the characteristics of the enzymes involved is highly important.

**Figure 1:**
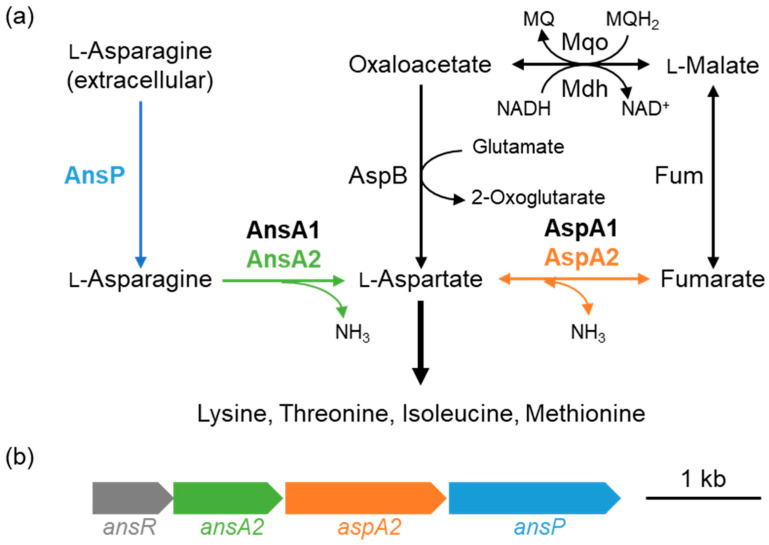
(**a**) Metabolic pathway of L-asparagine and L-aspartate in *Corynebacterium glutamicum* strain R. Proteins encoded by the *ans* gene cluster (**b**) are shown in the corresponding colors. AnsA1 and AnsA2: asparaginase; AspA1 and AspA2: aspartase; Fum: fumarase; AspB: L-aspartate aminotransferase; Mqo: malate:quinone oxidoreductase; Mdh: malate dehydrogenase; MQ: menaquinone; and MQH_2_: menaquinol. (**b**) The *ans* gene cluster.

Among the amino acids highly produced by *C. glutamicum*, L-lysine belongs to the aspartate family amino acids (L-Lys, L-Met, L-Thr, and L-Ile), which have a broad spectrum of applications, including those as food additives, feed supplements, pharmaceuticals, cosmetics, and polymer materials [4,7]. A number of strains have been developed for the production of the aspartate family amino acids based on findings from random mutagenesis and omics studies (reviewed in [7]). The precursor L-Asp is formed by aspartate aminotransferase, which catalyzes the amination of oxaloacetate using glutamate as an amino donor. The second pathway for L-Asp formation is the deamination of L-Asn by asparaginase. Endogenous asparaginase in *C. glutamicum* has been purified and characterized [8]. Aspartate ammonia-lyase (aspartase), which catalyzes the reversible deamination of L-Asp to fumarate and ammonium, is encoded in the genome of *C. glutamicum* and acts as the third pathway for supplying L-Asp (Figure 1a). However, a previous study reported no aspartase activity in *C. glutamicum* [9]. Even though asparaginase and aspartase are encoded in the genome, *C. glutamicum* has been recognized to be incapable of utilizing L-Asp and L-Asn as a carbon source.

Biochemical and genetic studies regarding the bacterial utilization of L-Asn and L-Asp have been reported. Two enzymes, namely asparaginase and aspartase, are required for the use of the amino acids (Figure 1). Asparaginase deaminates L-Asn to generate L-Asp and ammonia. Aspartase deaminates L-Asp to fumarate, which is metabolized in the TCA cycle. *Escherichia coli* has one aspartase and two asparaginases; one is a cytoplasmic enzyme with low affinity, while the other is located in the periplasm and has high affinity [10,11]. The periplasmic enzyme AnsB plays a role in the provision of an electron acceptor under anaerobic conditions [12]. In *Bacillus subtilis*, asparaginase and aspartase are encoded by the *ansAB* operon, which is induced in response to extracellular L-Asn [13]. As asparaginase releases ammonia from L-Asn, another asparaginase, AnsZ, is regulated in response to nitrogen limitation [14]. *Pseudomonas aeruginosa* utilizes L-Asn and L-Asp as carbon and nitrogen sources and D-Asn as a nitrogen source. It possesses two asparaginases and one aspartase, which are induced by L-Asn and L-Asp, respectively [15]. *Mycobacterium tuberculosis* has secretory asparaginase and uses the enzyme to assimilate nitrogen and to resist acid stress in macrophages [16]. In the enteric bacterium *Yersinia pseudotuberculosis*, aspartase is upregulated under acidic conditions and is involved in acid stress resistance [17]. Thus, carbon and nitrogen sources and environmental stress affect the expression of bacterial asparaginase and aspartase.

In this study, we identified and characterized a gene-cluster encoding asparaginase, aspartase, and L-Asn permease in the genome of *C. glutamicum* strain R. The gene products conferred the ability to utilize L-Asn as a carbon source. The expression of these genes was induced in response to exogenous L-Asn. The enzymes in this cluster would offer an alternative aspartate supply option for amino acid production.

## 2. Materials and Methods

### 2.1. Bacterial Strains, Plasmids, and Culture Conditions

The bacterial strains and plasmids used in our study are listed in Table 1. For genetic manipulation, *E. coli* strains were cultivated in lysogeny broth (LB) at 37 °C. For the growth experiments, *C. glutamicum* strains were precultured overnight at 33 °C in a nutrient-rich medium (A medium) [18] supplemented with 2% glucose. The cells were inoculated into a minimal medium (BTM medium) [19] supplemented with 2% glucose or 20 mM amino acids in 96-well titer plates. The cultivation and growth monitoring were performed using a microplate reader HiTS (Scinics, Tokyo, Japan) as described previously [20]. The optical density (OD) at 600 nm was monitored for 24 h. For expression analysis, *C. glutamicum* strains were precultured as described above. The cells were resuspended in fresh A medium and inoculated into 100 mL of A medium in a 500 mL flask. The initial OD at 610 nm was 0.5. After 3 h, L-Asn or L-Asp was added at 20 mM. Growth was monitored by measuring the OD at 610 nm. The antibiotic concentrations used for *E. coli* cultures included 50 μg ampicillin mL^−1^ and 50 μg kanamycin mL^−1^, and kanamycin (50 μg mL^−1^) was used in the case of *C. glutamicum*.

### 2.2. Construction of Plasmids and Mutants

General recombination experiments, including the transformation of *E. coli* and plasmid extraction, were performed according to standard protocols [21]. To construct the plasmids for the deletion of the target gene, the flanking regions of the gene were amplified using the primers listed in Table S1. The PCR fragments were fused by overlapping PCR or ligation and then cloned into the sucrose counter-selectable suicide vector pCRB725 [24]. Wild-type *C. glutamicum* cells were transformed using the constructed plasmids. Kanamycin-resistant clones were screened using sucrose medium, as described previously [24]. The *ansA1* gene in the *asnA2* deletion mutant or the *aspA1* gene in the *aspA2* deletion mutant was deleted to construct double-deletion mutants of asparaginase or aspartase genes.

### 2.3. RNA Extraction

For expression analysis, *C. glutamicum* strains were precultured overnight at 33 °C in A medium supplemented with 2% glucose. The cells were resuspended in fresh A medium and inoculated into 100 mL of A medium in a 500 mL flask (the initial OD at 610 nm was 0.5). After 3 h, L-Asn or L-Asp was added at 20 mM. For RNA stabilization, the sampled culture was mixed with an equal volume of RNAprotect Bacteria Reagent (Qiagen, Hilden, Germany). After incubation at room temperature, the cells were collected by centrifugation and stored at −80 °C. Total RNA was isolated using NucleoSpin^®^ RNA (MACHEREY-NAGEL, Düren, Germany) according to the manufacturer’s instructions. Purified RNA was treated with DNase I (Takara Bio Inc., Shiga, Japan), precipitated using ethanol, suspended in RNase-free water, and stored at −80 °C until further use.

### 2.4. Determination of Transcriptional Start Site (TSS)

The TSS of the *ans* operon was determined by 5′- rapid amplification of cDNA ends (RACE) analysis using the SMARTer™ RACE cDNA Amplification Kit (Clontech Laboratories, Inc., Mountain View, CA, USA), as described previously [25]. The total RNA extracted from the cells was poly(A)-tailed. cDNA was synthesized using the supplied oligo-dT anchored primer. The cDNA was amplified using the Universal Primer A (supplied with the kit) and gene-specific primer (Table S1). PCR products were cloned into the pGEM-T Easy Vector (Promega Corporation, Madison, WI, USA). Several clones harboring the 5ʹ RACE-PCR product were sequenced to determine the TSS.

### 2.5. Quantitative Reverse-Transcription Polymerase Chain Reaction (qRT-PCR)

qRT-PCR was performed using the 7500 Fast Real-Time PCR System (Thermo Fisher Scientific, Waltham, MA, USA) and Power SYBR^®^ Green PCR Master Mix with MuLV Reverse Transcriptase and RNase Inhibitor from the GeneAmp RNA PCR Kit (Thermo Fisher Scientific), as previously described [26]. The primers used are listed in Table S1. The comparative threshold cycle method (Thermo Fisher Scientific) was used to quantify the relative expression, and the relative expression ratios of each gene were normalized using the values for 16S rRNA.

### 2.6. Overexpression and Purification of His-Tagged AnsR

The *ansR* gene was amplified and cloned into pColdI (Takara) to generate pCRC667. *E. coli* BL21(DE3) cells were transformed with pCRC667 and grown at 37 °C in LB medium until OD at 600 nm reached 0.5. After cooling and incubating at 15 °C, 1 mM IPTG was added to induce His-tagged AnsR expression. Cells were collected and mechanically disrupted using FastPrep FP120 (Thermo Savant, Waltham, MA, USA). The His-tagged protein was purified with affinity chromatography using Ni-NTA agarose (Qiagen) according to the manufacturer’s instructions. The eluted protein was desalinated using a PD-10 column (GE Healthcare Bioscience, Piscataway, NJ, USA) pre-equilibrated with buffer (50 mM Tris-HCl pH 7.5, 10 mM MgCl_2_, and 1 mM EDTA). The protein concentration was determined using the Bio-Rad protein assay reagent (Bio-Rad Laboratories Inc., Hercules, CA, USA) with bovine serum albumin (BSA) as a standard.

### 2.7. Electrophoretic Mobility Shift Assay (EMSA)

DNA fragments encompassing the promoter and intragenic regions of the *ans* operon were generated by PCR using the primers listed in Table S1 and were used as DNA probes. The DNA probes were incubated with purified His-tagged AnsR in buffer (20 mM Tris-HCl pH 8.0, 100 mM NaCl, 3 mM MgCl_2_, 5 mM CaCl_2_, 0.1 mM EDTA, 0.1 mM DTT, 10% glycerol, and 0.15 mg/mL BSA) at room temperature for 25 min. To examine the effect of effector molecules on AnsR binding, the AnsR was incubated for 15 min with effectors prior to incubation with DNA probes. The reaction mixtures were loaded onto a 5% polyacrylamide gel and electrophoresed in 0.5x Tris-borate-EDTA buffer (89 mM Tris, 89 mM borate, and 2 mM EDTA, pH 8.3). The gel was stained with SYBR^®^ Green EMSA nucleic acid gel stain (Thermo Fischer Scientific, Waltham, MA, USA) and visualized using LAS3000 (Fujifilm, Tokyo, Japan).

## 3. Results

### 3.1. Conservation of the *ans* Gene Cluster

In the genome of *C. glutamicum* strain R, we found a gene cluster consisting of four genes (cgR_2807-cgR_2810); the genes encoded a GntR-type transcriptional regulator, asparaginase, aspartase, and permease, respectively (Figure 1b). We designated the genes in the cluster as *ansR*, *ansA2*, *aspA2*, and *ansP*, respectively. Another asparaginase, AnsA1, and aspartase, AspA1, were encoded by cgR_2025 and cgR_1563, respectively, at different loci in the genome. While *ansA1* encoding AnsA1 and *aspA1* encoding AspA1 are conserved among all corynebacterial genomes, a homologous gene cluster encoding asparaginase and/or aspartase is not conserved in the type strain ATCC 13032 but is found in the genomes of the following limited species of Corynebacterium: *Corynebacterium suranareeae*, *Corynebacterium deserti*, *Corynebacterium callunae*, and *Corynebacterium cyclohexanicum*. The function of the genes in the operon has not been investigated in any of the bacteria mentioned above.

### 3.2. Role of Genes in the Operon for L-Asn Utilization

As *ansA2* encoded an asparaginase, the utilization of L-Asn as a carbon source was examined. When L-Asn was used as the sole carbon source, strain R was able to grow, but strain ATCC 13032, in which the *ans* cluster was not encoded, was unable to grow (Table 2), suggesting the involvement of the gene cluster in L-Asn utilization. We examined the role of these genes in the *ans* cluster by individually deleting each gene. None of the deletion mutants of *ansA2*, *aspA2*, or *ansP* could grow in minimal medium with L-Asn as the sole carbon source, indicating that the genes in the cluster were essential for L-Asn utilization. In contrast, the deletion mutants of *ansA1* and *aspA1*, which encoded another asparaginase, AnsA1, and aspartase, AspA1, respectively, grew at the same level as that observed for the wild type, showing that these enzymes are not involved in L-Asn utilization.

To examine the role of the gene cluster in the utilization of L-Asn as a nitrogen source, the gene deletion mutants were cultivated in minimal medium containing glucose as a carbon source and L-Asn as a nitrogen source. All deletion mutants, including the *ansA1* and *aspA1* mutants, grew in the medium, indicating that the asparaginases AnsA1 and AnsA2 functioned equally in utilizing L-Asn as a nitrogen source. Indeed, double-deletion mutants of *ansA1* and *ansA2* were unable to utilize L-Asn as the nitrogen source (Table 1).

### 3.3. *ans* Operon Induced upon L-Asn Supplementation

**Figure 2:**
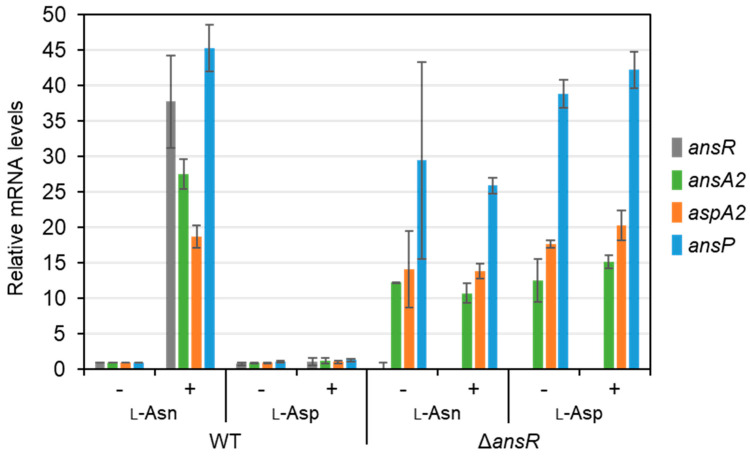
Expression analysis of the *ans* operon genes in response to the supplementation of L-asparagine or L-aspartate. RNA was extracted before (−) or 30 min after (+) the amino acid supplementation. Transcript levels of *ansR*, *ansA2*, *aspA2*, and *ansP* were determined by quantitative reverse-transcription polymerase chain reaction analysis. Transcript levels in the WT before the amino acid supplementation were standardized to 1. Mean values obtained from three independent cultivations are shown with their standard deviations.

The *ans* gene cluster was shown to be essential for L-Asn utilization. To examine whether the gene cluster was induced by L-Asn, the expression of the genes was measured before and after the supplementation of L-Asn. The wild-type strain was grown in nutrient-rich medium for 3 h, and then L-Asn or L-Asp was supplemented at 20 mM. The expression of the genes in the cluster was highly upregulated after 30 min of L-Asn supplementation (Figure 2). Supplementation of L-Asp had no effect on the gene expression. These results show that the *ans* gene cluster was induced in response to L-Asn.

**Figure 3:**
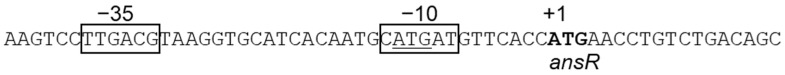
The promoter region of the *ans* operon. The transcriptional start site is indicated with +1. The initiation codon of *ansR* is shown in bold letters. The incorrect initiation codon previously deposited is indicated with an underline. The −35 and −10 regions of the σ^A^-dependent promoter are indicated using boxes.

These four genes were similarly upregulated, indicating that they form an operon. RT-PCR analysis using RNA extracted from cells grown in the presence of L-Asn demonstrated that these four genes were transcribed as a polycistronic mRNA. The transcriptional start site (TSS) was determined with RACE-PCR. The TSS was mapped to adenine at position +13 with respect to the initiation codon ATG of the first gene, *ansR*. As another ATG codon existed at this position, the original annotation was incorrect, and the gene cluster was transcribed as a leaderless mRNA from the adenine of the newly defined initiation codon (Figure 3). The −10 and −35 regions of the σ^70^-type housekeeping sigma factor σ^A^-dependent promoter [27] were located upstream of the TSS (Figure 3).

### 3.4. *ans* Operon Repressed by AnsR

The first gene of the operon, *ansR,* encoded a GntR-type transcriptional regulator. To examine whether the regulator was involved in the L-Asn-responsive expression of the operon, an *ansR* gene deletion mutant was constructed. The wild type and *ansR* deletion mutant were cultivated in nutrient-rich medium and supplemented with L-Asn or L-Asp to examine the changes in the expression of the *ans* operon, as described above. The expression of the operon genes in the deletion mutant was much higher than that in the wild type before the supplementation of amino acids (Figure 2). The expression levels of the genes in the mutant were maintained for 30 min with L-Asn or L-Asp supplementation. In the wild type, the expression of the *ans* operon was not altered upon supplementation of L-Asp. These findings indicate that the *ans* operon was repressed by AnsR. The growth of the *ansR* deletion mutant on nutrient-rich medium supplemented with L-Asp was comparable to that of the wild type, whereas the mutant reached a slightly higher OD than the wild type in the growth experiments carried out using medium supplemented with L-Asn.

### 3.5. AnsR Directly Repressed the *ans* Operon

**Figure 4:**
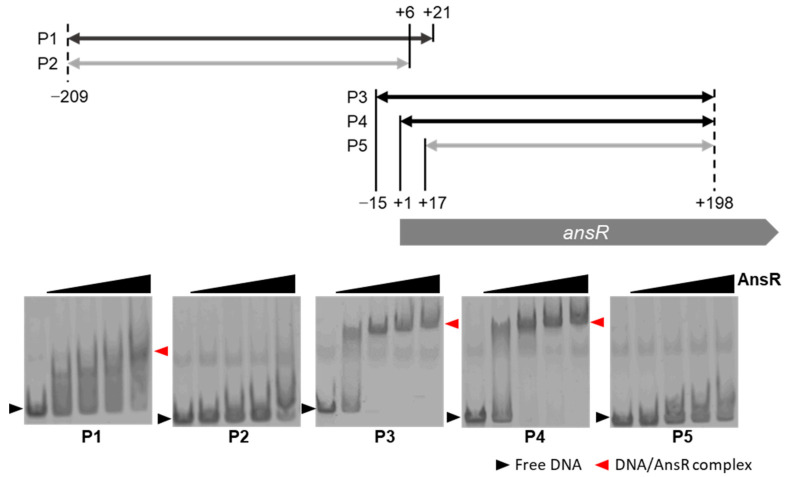
Electrophoretic mobility shift assay using His-tagged AnsR. The DNA probes (P1 to P5) encompassing the upstream or intragenic region of the *ansR* gene are indicated. The DNA probes interacting with AnsR are indicated in black, while those without interaction are indicated in grey. The DNA probes (10 nM) were incubated with varying amounts of His-tagged AnsR (0.2–1.6 μM). The free probes and AnsR-DNA complexes are indicated with black and red arrowheads, respectively.

To examine whether AnsR directly represses the *ans* operon, the binding of AnsR to the promoter region of the operon was investigated using EMSA. A DNA probe encompassing the promoter region (positions −209 and +21 with respect to the TSS) was incubated with the purified AnsR. A shifted band was observed, which increased in an AnsR-amount-dependent manner (Figure 4). Therefore, it was concluded that AnsR binds to the promoter region and directly represses operon expression.

**Figure 5:**
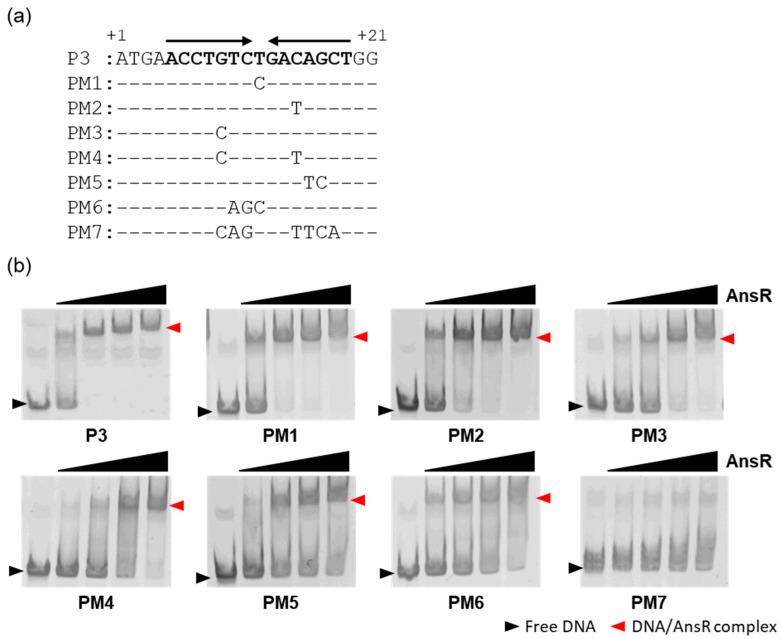
Mutational analysis of the AnsR binding site. (**a**) The sequences of the used DNA probes are depicted. Mutations were introduced into the PM probes, and only exchanged nucleotides are shown. The binding site is shown in bold letters and indicated with arrows. The position with respect to the transcriptional start site of *ansR* is indicated. (**b**) Electrophoretic mobility shift assay using the mutated DNA probes. The DNA probes (10 nM) were incubated with varying amounts of His-tagged AnsR (0.2–1.6 μM). Free probes and AnsR-DNA complexes are indicated with black and red arrowheads, respectively.

To locate the AnsR binding site, a series of DNA probes encompassing the upstream and intragenic regions of the *ansR* gene were used in the EMSA. AnsR did not interact with the DNA probe encompassing the region between positions −209 and +6 with respect to the TSS, but it bound to the DNA probe encompassing positions +1 and +198 (Figure 4). Combined with these findings, the region between positions +1 and +21 with respect to the TSS is likely to be required for AnsR binding. In this region, the inverted repeat 5′-ACCTGTCTGACAGCT-3′ was found at positions +5 and +19 with respect to the TSS. To investigate the involvement of this site in AnsR binding, EMSA was performed with DNA probes containing mutations in the putative binding site (Figure 5). As the putative binding site was located in the intragenic region, the mutation(s) was/were designed to make synonymous codons, except for probe PM7. A DNA probe containing mutations in the inverted repeat reduced the affinity for AnsR. In contrast, the point mutation of T at the center of the binding site had no effect on the binding. The DNA probe with the inverted repeat that was completely exchanged using several mutations (PM7) had entirely lost its interaction with AnsR. These findings confirm that the inverted repeat was the AnsR-binding site. The finding that AnsR binding to the P1 probe was weaker than its bindings to the P3 and P4 probes (Figure 4) is likely because the binding site was located at the end of the P1 probe.

**Figure 6:**
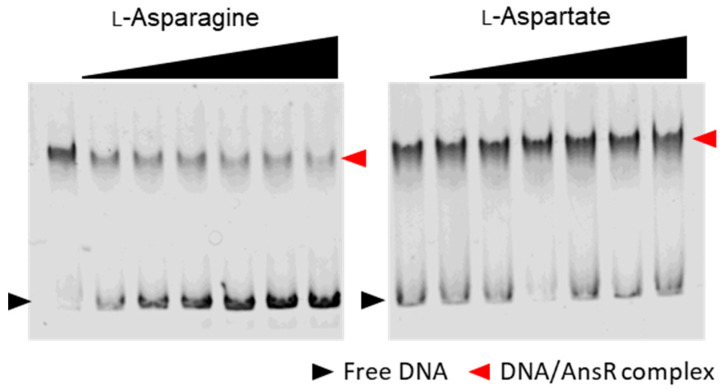
Investigation of effector molecules of AnsR. AnsR was incubated with L-asparagine or L-aspartate prior to the incubation with the DNA probe. The concentrations of the amino acids were 0, 0.2, 0.4, 0.8, 1.0, 2.0, and 5.0 mM. Free probes and AnsR-DNA complexes are indicated with black and red arrowheads, respectively.

To search for metabolites (effector molecules) affecting AnsR binding, AnsR was incubated with L-Asn or L-Asp prior to incubation with the DNA probe. When AnsR was incubated with the DNA probe in the presence of L-Asn, free DNA increased in a dose-dependent manner, indicating that L-Asn inhibited the binding of AnsR to the site (Figure 6). In contrast, L-Asp had no effect on AnsR binding.

## 4. Discussion

In this study, we identified and characterized the *ans* operon that encodes asparaginase, aspartase, and L-Asn transporter in *C. glutamicum*. Gene deletion studies have revealed that the genes in this operon are essential for L-Asn utilization as a carbon source. Other asparaginase and aspartase, which are presumably and constitutively expressed, are not involved in L-Asn utilization. Expression analysis demonstrated that the genes in the operon are co-transcribed and are induced by L-Asn. L-Asn-responsive expression is controlled by the transcriptional regulator AnsR, whose DNA-binding activity is inhibited in the presence of L-Asn.

The proteins encoded in the operon showed the highest identity with those in the genomes of bacteria of the genera *Arthrobacter*, *Glutamicibacter*, and *Rhodococcus*, which belong to the class Actinobacteria, and the genus *Rhizobium* of Alphaproteobacteria. To the best of our knowledge, L-Asn utilization genes have not been demonstrated in the bacteria belonging to the Actinobacteria class described above. Thus, the findings related to the *ans* operon in *C. glutamicum* provide insights into the physiological functions and regulation of the genes in related bacteria. For example, *Mycobacterium dioxanotrophicus*, which belongs to the class Actinobacteria, possesses the AnsR ortholog BTO20_32665. L-Asn permease is encoded directly downstream of this gene, suggesting that the permease is repressed by the regulator, although neither asparaginase nor aspartase gene are found in the flanking region.

The *ans* operon is repressed by AnsR under normal growth conditions and derepressed in response to the presence of L-Asn. L-Asn-responsive transcriptional regulatory system for the asparaginase gene is prevalent in bacteria. *Rhizobium etli* contains a similar gene cluster encoding a GntR-type transcriptional regulator (AnsR), L-Asn permease (AnsP), asparaginase (AnsA), and aspartase (AnsB) in that order, in the large plasmid p42e [28,29]. Unlike the *ans* operon in *C. glutamicum*, the transcriptional regulator gene is not co-transcribed with the other genes *ansPAB* [28]. Indeed, the σ^70^-type promoter motif is found between *ansR* and *ansP* [28,30]. Although not directly demonstrated, the transcriptional regulator AnsR is involved in L-Asn-responsive induction of this operon [28]. The expression of the operon was not induced by L-Asp. The expression level induced by L-Asn was decreased in the presence of other carbon sources such as glucose and glycerol [31]. The expression of the *Rhizobium* operon was insensitive to the nitrogen source in the medium as well as oxygen levels. The operon *ansAB* encoding the asparaginase AnsA and the aspartase AnsB in *B. subtilis* is also induced in response to the presence of L-Asn. The XRE-family transcriptional regulator AnsR, which is encoded by *ansR* and divergently transcribed from the operon, represses the *ansAB* operon and mediates L-Asn-responsive expression [13,14,32]. *B. subtilis* has another asparaginase, AnsZ, which is positively regulated by the global nitrogen regulator TnrA in response to a nitrogen source [14]. While both asparaginases AnsA and AnsZ liberate ammonia, their expression is regulated by distinct regulators that sense different molecules.

In *P. aeruginosa*, the L-Asn permease AnsP and asparaginase AnsA are encoded by the *ansPA* operon, which is induced in response to L-Asn [15]. The Cro/CI family transcriptional regulator AnsR, which is divergently transcribed from *ansP*, positively regulates this operon. In addition, the aspartate ammonia lyase (aspartase) AspA is encoded in a different genomic locus and is positively regulated by the LysR-type transcriptional regulator AspR in response to extracellular or endogenous L-Asp [15]. In addition to *aspA*, transporters for acidic amino acids and C4 dicarboxylate, which are induced in response to L-Asp, are required for the amino acid utilization. *C. glutamicum* is unable to utilize L-Asp as a carbon source, probably because of the lack of a corresponding uptake system.

To the best of our knowledge, *C. glutamicum* AnsR is the first case of an asparagine catabolic gene repressor whose binding site has been identified. The AnsR binding site is located downstream of the TSS, indicating a roadblock mechanism for repression by AnsR. AnsR belongs to the FadR subfamily of the GntR family, whose consensus binding site is 5′-TNGTNNNACNA-3′ [33]. The identified AnsR binding site was the inverted repeat 5′-ACCTGTCTGACAGCT-3′, which partially matched the consensus site. Mutational analysis of the binding site confirmed the importance of the inverted repeat. Highly similar putative binding sites are found in the 5′-region of the AnsR encoding genes in the bacteria of the genera *Arthrobacter*, *Glutamicibacter*, and *Streptomyces*, which carry the orthologous asparagine operon in the genome. This is likely because the six N-terminal residues of orthologous regulators in these bacteria are highly conserved. In the genome of some species of the genus *Rhodococcus* in the Actinobacteria class, the AnsR encoding gene is divergently transcribed from genes encoding aspartase (AspA), L-Asn permease (AnsP), and asparaginase (AnsA). A putative AnsR binding site was found in the intergenic region between *ansR* and *aspA*. Thus, the operon in these bacteria is plausibly controlled by a comparable transcriptional regulatory system.

Aspartase catalyzes not only the deamination of aspartate to fumarate for utilization as a carbon source, but also the amination of fumarate to L-Asp with ammonia (Figure 1a). Generally, L-Asp is synthesized by aspartate aminotransferase, which catalyzes oxaloacetate amination using L-Glu as an amino donor. The amination reaction of aspartase, which utilizes ammonia instead of L-Glu, could be applied to the production of the aspartate family amino acids. Indeed, to increase L-Asp availability for L-Lys production, *E. coli* aspartase has been introduced into a L-Lys production strain of *C. glutamicum*. When fumarate was supplied, L-Lys production was increased [9]. The enzymatic characterization of the aspartases AspA1 and AspA2 is worth investigation for the application.

The *ans* operon is encoded in limited corynebacterial species and strains. Similarly, the strain-specific genes involved in carbon utilization and outer protein synthesis have been identified in *C. glutamicum*: β-glucoside [34,35], shikimate [36], phenylacetate [37], and cellular surface protein [38]. Characterization of such strain-specific genes would advance our understanding of the catabolic capability of *C. glutamicum* and lead to the identification of novel regulatory mechanisms.

## Figures and Tables

**Table 1:** Bacterial strains and plasmids used in this study.

Strain or Plasmid	Relevant Characteristics	Source or Reference
Strains		
*E. coli*		
JM109	*recA1 endA1 gyrA96 thi hsdR17 supE44 relA1* Δ(*lac-proAB*)/F’[*traD36 proAB*^+^ *lacI^q^ lacZ*ΔM15]	Takara
JM110	*dam dcm supE44 hsdR17 tih leu rpsL lacy galK galT ara tonA thr tsx* Δ(*lac-proAB*)/F’[*traD36 proAB^+^ lacI^q^ lacZ*ΔM15]	[21]
BL21(DE3)	F^−^ *ompT gal dcm lon hsdS*_B_(r_B_^−^ m_B_^−^) λ(DE3)	[22]
*C. glutamicum*		
R (JCM 18229)	Wild-type strain	[23]
ATCC 13032	Wild-type strain	American Type Culture Collection, Manassas, VA, USA
ΔaspA1	R with deletion in *aspA1*	This study
ΔansA1	R with deletion in *ansA1*	This study
ΔansR	R with deletion in *ansR*	This study
ΔansA2	R with deletion in *ansA2*	This study
ΔaspA2	R with deletion in *aspA2*	This study
ΔansP	R with deletion in *ansP*	This study
ΔaspA1A2	ΔaspA2 with deletion in *aspA1*	This study
ΔansA1A2	ΔansA2 with deletion in *ansA1*	This study
Plasmids		
pCold	Apr: a vector for cold-inducible expression	TaKaRa
pCRA725	Km^r^; the suicide vector containing the *B. subtilis sacB* gene	[24]
pCRC667	Ap^r^: pColdI with a coding region of the *ansR* gene	This study
pCRC668	Km^r^; pCRA725 with a fragment containing flanking regions of *aspA1*	This study
pCRC669	Km^r^; pCRA725 with a fragment containing flanking regions of *ansA1*	This study
pCRC670	Km^r^; pCRA725 with a fragment containing flanking regions of *ansR*	This study
pCRC671	Km^r^; pCRA725 with a fragment containing flanking regions of *ansA2*	This study
pCRC672	Km^r^; pCRA725 with a fragment containing flanking regions of *aspA2*	This study
pCRC673	Km^r^; pCRA725 with a fragment containing flanking regions of *ansP*	This study

**Table 2:** Growth properties of strains on glucose or amino acids as a sole carbon or nitrogen source. Cell growth was monitored using optical density at 600 nm for 24 h. Data of deletion mutants of genes in the *ans* operon are highlighted with the same color as the corresponding genes in Figure 1. Data of type strain ATCC 13032 are highlighted with gray.

Genotype	Carbon or Nitrogen Source		Amino Acids As
	Glucose	L-Asparagine	
R (Wild type)	++ ^1^	++	Carbon
		++	Nitrogen
ATCC 13032	++	− ^1^	Carbon
		−	Nitrogen
Δ*aspA1*	++	++	Carbon
		++	Nitrogen
Δ*aspA2*	++	−	Carbon
		+ ^1^	Nitrogen
Δ*aspA1*Δ*aspA2*	++	−	Carbon
		−	Nitrogen
Δ*ansA1*	++	++	Carbon
		++	Nitrogen
Δ*ansA2*	++	−	Carbon
		++	Nitrogen
Δ*ansA1*Δ*ansA2*	++	−	Carbon
		−	Nitrogen
Δ*ansP*	++	−	Carbon
		+	Nitrogen

^1^ ++ indicates wild-type growth, + indicates slower growth, − indicates no growth.

## Supplementary Materials

The following are available online at https://www.mdpi.com/article/10.3390/microorganisms10051002/s1, Table S1. Oligonucleotide primers used in this study.
